# Edible Bird Nest Supplementation Enhances Male Reproductive Function: Current Insights and Future Horizons

**DOI:** 10.3390/foods14213759

**Published:** 2025-11-01

**Authors:** Farah Hanan Fathihah Jaffar, Nurul Atiqah Azhar, Sofwatul Mokhtarah Maluin, Khairul Osman, Siti Fatimah Ibrahim

**Affiliations:** 1 Department of Physiology, Faculty of Medicine, Universiti Kebangsaan Malaysia, Level 18, Preclinical Building, Jalan Yaacob Latif, Cheras, Kuala Lumpur 56000, Malaysia; 2 Department of Physiology, Faculty of Medicine and Health Sciences, Universiti Sains Islam Malaysia, Persiaran Ilmu, Putra Nilai, Nilai 71800, Negeri Sembilan, Malaysia; 3 Center for Diagnostics, Therapeutics, and Investigative Studies (CODTIS), Faculty of Health Sciences, Universiti Kebangsaan Malaysia, Jalan Raja Muda Abdul Aziz, Kuala Lumpur 50300, Malaysia; khairos@ukm.edu.my

**Keywords:** EBN, reproductive hormones, spermatogenesis, male infertility, proliferation, antioxidant

## Abstract

Edible bird nest (EBN) has a longstanding tradition in Chinese herbal medicine as a natural supplement for enhancing health and well-being. Current scientific research indicates that EBN possesses properties conducive to improving male reproductive function. These properties include promoting cell proliferation, containing essential reproductive hormones, and exhibiting antioxidant activity. Despite these promising characteristics, the potential of EBN’s specific effects on male reproductive health, particularly in addressing infertility, remains sparsely discussed. Hereby, this review aims to present the available scientific evidence, discuss potential benefits, and underscore the importance of future research in advancing our understanding of EBN’s role in male reproductive health. This work is imperative to expand our knowledge of the biological underpinnings of EBN so that it can be developed as a food supplement and alternative treatment for male infertility.

## 1. Introduction

Edible bird nest (EBN) has been valued in traditional Chinese medicine as a food supplement with diverse health-promoting effects. It is produced from the saliva of male swiftlets during the breeding season [1]. About 24 swiftlet species have been recognised globally and grouped into four genera. Among these, *Hydrochous*, *Schoutedenapus*, and *Collocalia* are non-echolocating swiftlets, while the genus *Aerodramus* is echolocating. The white nest produced by *Aerodramus fuciphagus* is the most common and commercially important type in Southeast Asia [2,3,4].

Research on EBN began more than two centuries ago. Early studies on EBN primarily focused on the nature of the nest, including the materials used in its construction [5], and the glandular source of the mucus that binds the nest [6,7]. Over time, research interest shifted towards its nutritional composition. Studies have established that EBN predominantly comprises protein followed by carbohydrates, fat, ash, and trace moisture [8,9,10]. It also contains essential minerals such as sodium and calcium, which are in higher concentrations than magnesium, potassium, phosphorus and iron, and amino acids necessary for physiological functions [10,11]. This distinctive nutritional profile has driven further research into its bioactive and therapeutic potential.

Traditionally, EBN has been credited with multiple health benefits, including improving digestion, easing asthma, dissolving phlegm, and promoting the speedy recovery of illnesses, surgery, and wounds [2,12]. It has also been associated with enhanced complexion, delayed ageing process, improved voice and attention, and increased libido [2,12].

Scientific studies have begun to validate these claims, and various reports showed that EBN possesses antimicrobial and antiviral properties [13,14], anti-inflammatory effects [15,16,17], antioxidant activity [17,18,19], and anti-ageing potential [20]. In addition, EBN has been reported to exert anti-osteoarthritis effects [21,22], enhance wound healing [23], and promote cell proliferation [15,24,25]. In vivo studies further support its therapeutic value, demonstrating improvements in osteoporosis [26], anti-diabetic activity [27], and enhancing spatial learning and memory [28,29].

Among the many reported benefits, EBN is increasingly recognised for its potential role in reproductive health. Traditional claims suggest that EBN enhances libido [2,12,30]. Libido is a term used to describe a sexual drive or desire that involves a complex interaction involving steroid hormones, motivating external environment, and cognitive function [31]. However, whether the libido in the traditional claim of EBN refers to one or both sexual partners is unclear. Nevertheless, the reproductive function requires more than a sexual urge. The significant component of reproductive function is to produce gametes so that fertilisation and subsequent pregnancy can occur.

Interestingly, specific properties of EBN are directly relevant to the nature of the reproductive system. Its cell-proliferative activity [15,24,25] parallels the cellular processes involved in gametogenesis. EBN also contains vital hormones [12] crucial to the reproductive system’s physiological function. Additionally, its antioxidant activity [17,18,19] protects gametes from oxidative stress, a key factor in gamete integrity.

Despite these promising features, their implications for male reproductive health have been poorly discussed. Therefore, this review comprehensively discussed characteristics of EBN in the male reproductive system. Additionally, this review aimed to compile all recent scientific information supporting EBN’s ability to improve various conditions related to the male reproductive system. This evaluation could serve as a platform for EBN’s potential as a food supplement to enhance the reproductive system, particularly as the world’s fertility crisis gains momentum.

## 2. Nutritional Composition of EBN

Edible bird’s nest (EBN) is valued not only for its cultural significance but also for its rich nutritional profile. Proteins comprise the most significant portion of their content, followed by carbohydrates, ash, fat, and a small amount of moisture [8,9,10,11]. Alongside these macronutrients, EBN provides a wide variety of amino acids, both essential, such as lysine, threonine, leucine, and valine, and non-essential, including serine, glutamic acid, proline, and tyrosine [9,32]. Some amino acids, like tyrosine and glutamic acid, have even been proposed as markers to distinguish nests from different sources [33]. These amino acids play vital roles in reproductive physiology, as glutamic acid contributes to spermatogonial cell energy metabolism [34]. Meanwhile, proline and lysine are essential substrates for collagen synthesis that contribute to the structural integrity of tissues, including the seminiferous tubules that support spermatogenesis [35].

EBN is also a source of complex carbohydrates, particularly in the form of glycoproteins. These include sialic acid and several oligosaccharides such as D-mannose, galactose, N-acetylgalactosamine (GalNAc), N-acetylglucosamine (GlcNAc), and N-acetylneuraminic acid (NANA) [32,36]. These glycans may support male reproductive function by facilitating cell–cell recognition, sperm–oocyte interaction, and protection of sperm membranes from oxidative damage [37].

In addition, EBN contains minerals, most notably sodium and calcium in higher amounts than magnesium, potassium, phosphorus, and iron [10,11]. Calcium, in particular, is essential for sperm motility and acrosome reaction [38], while magnesium and zinc are known cofactors in spermatogenesis and testosterone biosynthesis [39].

Interestingly, reproductive hormones, including testosterone, estradiol (E2), progesterone, follicle-stimulating hormone (FSH), luteinizing hormone (LH), and prolactin, have also been identified in EBN extracts [40]. Though present in small amounts, these hormonal components may contribute to its traditional reputation for improving vitality and reproductive health.

This diverse composition provides not only basic nutrition but also bioactive compounds that may synergistically support male reproductive physiology through energy provision, antioxidant protection, hormonal modulation, and spermatogenic maintenance. A detailed summary of its major constituents, including proteins, carbohydrates, amino acids, minerals, and vitamins, is presented in Table 1.

## 3. Characteristics of EBN Attributed to Male Reproductive Function

The beneficial effects of EBN on male reproductive health can be understood through three major characteristics that directly support reproductive function: (1) its proliferative effect on germ cells, (2) the presence of reproductive hormonal content, and (3) its potent antioxidant activity.

### 3.1. Proliferative Effect

EBN has been shown to exhibit biological activity similar to epidermal growth factor (EGF) [44], a single-chain polypeptide of 53 amino acids first described by Stanley Cohen in 1962 [45]. Since the discovery of EGF-like activity in EBN, multiple approaches have been employed to investigate its proliferative potential using different cell models.

Supplementation of processed and unprocessed EBN at five ppm promoted significant proliferation of human colonic adenocarcinoma (Caco-2) cells [24]. While the proliferative effect was initially attributed to EGF, further evidence suggested that sialic acid, GalNAc, and GlcNAc, the primary carbohydrate moieties in EBN, may also contribute to this characteristic. These glycans are known to mediate cell–cell and carbohydrate–protein interactions as well as downstream signalling [45,46], which are critical for cell proliferation.

EBN’s proliferative effects were observed through various in vitro studies. For instance, EBN supplementation accelerated the proliferation of spleen B-cells, while T-cells showed little response [47]. Furthermore, EBN was added as a serum replacement at varying concentrations to human adipose-derived stem cells (hADSCs), normal human fibroblasts (NHFs), and cancer cell lines (MCF-7 and Hep2B). At 2000 ppm, EBN increased proliferation of hADSCs and NHFs by 34% and 38%, respectively. In the meantime, no proliferative effect on cancer cell lines was recorded [25].

The differing results observed between Caco-2 [24] and other cancer cell lines [25] suggest that EBN’s proliferative effects may depend on factors such as cell type, EBN concentration, type and source of EBN, as well as the EBN doses applied under different experimental conditions. Although EBN tends to promote cell growth in normal cells, these findings should be interpreted with caution. Current evidence is not yet sufficient to confirm that EBN selectively supports normal cell proliferation or that it is inherently safe in relation to cancer risk. Therefore, further in vivo studies are essential to clarify its biological effects, including detailed dose–response evaluations, long-term exposure assessments, and comprehensive toxicological testing to ensure the safety of EBN consumption in humans.

Nevertheless, the proliferative effect of EBN appears to involve activation of the activator protein-1 pathway (AP-1) and nuclear factor kappa B (NF-κB), which in turn stimulate production of interleukin-6 (IL-6) and vascular endothelial growth factor (VEGF) through the p44/42 and p38 mitogen-activated protein kinase (MAPK) pathways [20,25].

**Figure 1:**
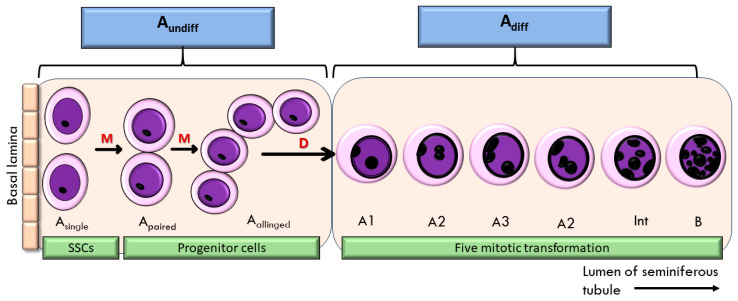
Proliferation of spermatogonia stem cells (SSC) in the seminiferous tubule of the testis. M—mitosis; D—differentiation; A_undiff_—undifferentiated spermatogonia; A_diff_—differentiated spermatogonia.

The relevance of these findings to male reproduction lies in the fact that spermatogenesis depends heavily on cell proliferation within the testis. Spermatozoa are produced in the seminiferous tubules, where spermatogonia stem cells (SSCs) undergo mitosis to maintain their pool and generate progenitors for spermatogenesis [48]. SSCs can divide symmetrically to produce identical daughter cells or asymmetrically to yield one SSC and one progenitor. These divisions give rise to A_paired_ and A_aligned_ spermatogonia, collectively referred to as undifferentiated spermatogonia [49]. Upon differentiation, these cells progress through successive mitotic divisions (A_1_–A_4_, intermediate, and B spermatogonia) before entering meiosis as spermatocytes [50] (Figure 1). This highly regulated process is controlled by Sertoli cells and influenced by growth factors, such as glial cell line-derived neurotrophic factor (GDNF), fibroblast growth factor (FGF), and EGF, which activate signalling cascades including Ras-ERK, PI3K/AKT, and MAPK pathways [51,52]. Given that EBN has been shown to contain EGF and to activate MAPK signalling [25], its supplementation may support spermatogonia proliferation and enhance spermatogenesis.

Beyond growth factor-like activity and carbohydrate moieties, EBN also contains vitamin A [36], an essential factor for the transition of A-aligned spermatogonia into differentiating A_1_ spermatogonia. The active derivative of vitamin A, retinoic acid (RA), regulates this transition through receptor phosphorylation. In the absence of RA, spermatogonia differentiation and subsequent meiosis cannot proceed [53,54]. While no studies have yet examined whether RA derived from EBN directly influences germ cell proliferation in the testis, the presence of this nutrient provides an additional plausible mechanism through which EBN may support spermatogenesis.

Taken together, EBN supplementation may enhance SSC proliferation through multiple complementary mechanisms: (i) growth factor-like activity, particularly via EGF and MAPK signalling; (ii) carbohydrate-mediated cell signalling; and (iii) provision of vitamin A/RA to drive spermatogonia differentiation.

In summary, both in vitro and in vivo evidence demonstrate that EBN can stimulate proliferation while remaining safe for non-cancerous cells. Its ability to enhance proliferation and differentiation through MAPK and RA-related mechanisms, and its protective effects against environmental and chemical insults, highlight EBN’s potential role in supporting spermatogenesis and addressing oligospermia-related male infertility.

### 3.2. Reproductive Hormonal Content

The physiological function of the male reproductive system is tightly regulated by reproductive hormones, particularly FSH, LH, and testosterone. FSH acts on Sertoli cells to promote spermatogonia proliferation and initiate spermatogenesis [55], activating multiple signalling pathways, including cAMP/PKA, MAPK, calcium, phosphoinositide 3-kinase (PI3K), and phospholipase A2 [56].

It also regulates retinoic acid signalling to stimulate spermatogonia differentiation [57]. On the other hand, LH stimulates Leydig cells to produce testosterone, which is essential for spermatogonia development, meiosis, and spermiation [58,59].

In addition, progesterone receptors, although the hormone is often regarded as female-specific, are also present in the seminal vesicles and testes [60]. Similarly, estradiol receptors α (ERα) and β (ERβ) have been identified in germ cells and along the spermatic ducts, highlighting the importance of estrogen in testicular function [61]. Meanwhile, evidence suggests that both estradiol and prolactin have been shown to influence spermatogenesis through the hypothalamic–pituitary–gonadal (HPG) axis and to contribute to the regulation of libido and erectile function [62,63].

In line with these physiological roles, EBN has been shown to contain several reproductive hormones, including testosterone, E2, progesterone, LH, FSH, and prolactin [12]. Advanced profiling using Orbitrap LC–MS analysis further confirmed the presence of testosterone, E2, and progesterone in EBN extracts [64]. For this EBN hormonal content, it is essential to note that these hormones are present only in trace concentrations. In addition, its bioavailability following oral consumption remains uncertain. Digestion and absorption processes may further limit their capacity to influence systemic hormone levels in humans.

Nevertheless, functional studies support the presence of these hormonal contents in EBN extract. For instance, EBN administration increased serum testosterone and LH in castrated male Wistar rats [40]. Meanwhile, another study observed a non-significant dose-dependent increase in serum testosterone, FSH, and LH in male Sprague Dawley rats [64]. Furthermore, 8 weeks of EBN supplementation in female rats significantly elevated E2, progesterone, and prolactin, with the highest dose (120 mg/kg/day) producing the most pronounced effects [65]. All these studies administered EBN via oral or intragastric routes.

Collectively, these findings suggest that EBN consumption may influence systemic hormone levels. In the context of male reproductive physiology, alterations in circulating hormone concentrations could, in turn, affect spermatogenesis and the HPG feedback loop. For instance, if systemic testosterone levels rise to a physiologically active range following EBN supplementation, this may suppress local intratesticular testosterone synthesis through negative feedback on gonadotropin release [66]. Such suppression could potentially impair spermatogenesis, as locally produced testosterone within the testis is essential for Sertoli cell function and germ cell development [67].

Therefore, while the hormonal components of EBN may contribute to reproductive regulation, their effects must be carefully balanced to ensure they do not disrupt the physiological homeostasis required for optimal sperm production and function. However, since these observations are based solely on animal studies, further evaluation is needed to determine whether such effects are physiologically significant in humans.

Although the systemic relevance of these hormonal effects remains uncertain, the presence of these reproductive hormones in EBN may partly explain the longstanding traditional claims that EBN enhances libido. However, its influence on libido remains hypothetical, as no direct evidence from animal or human studies is currently available. Further experimental and clinical investigations are needed to determine whether EBN supplementation has any measurable effect on sexual desire or performance.

Beyond this cultural perspective, the hormonal content of EBN highlights its potential as a supportive intervention for male infertility associated with hormonal imbalances. By influencing key regulators such as testosterone, LH, and FSH, EBN supplementation could help restore hormonal homeostasis and improve reproductive function. Nevertheless, its use should be approached cautiously in healthy men without infertility, as unnecessary exposure to exogenous hormones may carry unintended risks.

### 3.3. Antioxidant Activity

Its antioxidative activity represents the third prominent characteristic of EBN relevant to male reproductive function. The antioxidative activity of EBN was first demonstrated when its supplementation protected HEPG2 cells against toxicity induced by hydrogen peroxide (H_2_O_2_). Moreover, it exhibited free radical-scavenging capacity in 2,2′-azino-bis [3-ethylbenzothiazoline-6-sulphonic acid] (ABTS) and oxygen radical absorbance capacity (ORAC) assay [18].

Consistently, EBN prevented SH-SY5Y cells exposed to neurotoxin 6-hydroxypamine (6-OHDA), a model to imitate the onset of Parkinson’s disease [19]. Furthermore, EBN supplementation showed an increase in the expression of hepatic antioxidant genes, including superoxide dismutase (SOD) 1 and 2, glutathione reductase (Gsr), and glutathione peroxidase (Gpx), in a dose-dependent manner in rats that were fed with a high-fat diet [17].

Several EBN constituents appear to mediate these antioxidant effects. A comparison of the amino acid profile between EBN and chicken eggs showed that EBN demonstrated stronger 1,1-diphenyl-2-picrylhydrazyl (DPPH) and ABTS, primarily attributed to its high content of histidine, proline, phenylalanine, and tryptophan [68]. In addition, two pentapeptides derived from EBN hydrolysates, including Pro-Phe-His-Pro-Tyr and Leu-Leu-Gly-Asp-Pro, also demonstrated high ORAC value and could significantly protect HEPG2 cells from H_2_O_2_-induced oxidative damage [69]. These findings support the notion that the protein-rich composition of EBN is a valuable natural source of antioxidants [70].

In addition, bioactive compounds such as ovotransferrin, lactoferrin [71], sialic acid [72], and several vitamins, including vitamins A, D, and C, may also contribute to EBN’s antioxidative activity [73]. Thus, current evidence suggests that EBN exerts its antioxidant effects mainly through non-enzymatic mechanisms, although whether it also engages enzymatic antioxidant pathways remains to be clarified.

Numerous reports have linked male infertility to an increase in reactive oxygen species (ROS) and a decrease in antioxidant levels, a condition known as oxidative stress [74,75]. Elevated ROS levels were reported to impair sperm motility through the increase in oxidative stress in the mitochondria of asthenozoospermic cases [76,77]. The decreased seminal antioxidant levels, on the other hand, were associated with increased single DNA breaks in teratozoospermic instances [78]. Additionally, specific diseases like varicocele [79], cryptorchidism [80], and the presence of bacteria and/or leukocytes in semen [81] are also connected to elevated ROS levels.

Given the growing recognition of oxidative stress in male infertility, numerous antioxidants have been investigated. This includes vitamin E [82], ascorbic acid [83], coenzyme Q10 [84], lycopene [85], and melatonin [86]. Various natural products were also evaluated, and this includes honey [87,88,89], curcumin [90], and broccoli [91]. Most of the studies employed oral supplementation [84,85,86,87,88,89] to assess the antioxidant properties of the abovementioned natural products and substances in clinical and animal studies. However, some studies used different approaches, such as adding to drinking water [92] or using a substrate in sperm preparation medium [90,93].

These approaches could be adapted to evaluate the antioxidative potential of EBN to mitigate oxidative stress in the male reproductive system. Oral administration remains the most practical method, consistent with its traditional consumption as soup, and is increasingly supported by modern formulations such as energy drinks [30]. Following supplementation, the level of oxidative stress in seminal plasma and its effect on the quality of sperm for clinical research subjects should be assessed. In animal research, a comprehensive assessment can be carried out by evaluating the oxidative status of blood serum and testicular homogenate, assessing testicular tissue sections by using proper antioxidant/oxidant probes, and analysing the gene expression levels of any relevant antioxidant system in the testis. As oxidative stress is a major contributor to male infertility, the presence of natural antioxidants in EBN further underscores its potential as a dietary supplement to promote male reproductive health, particularly in modern contexts where oxidative stress is prevalent.

**Figure 2:**
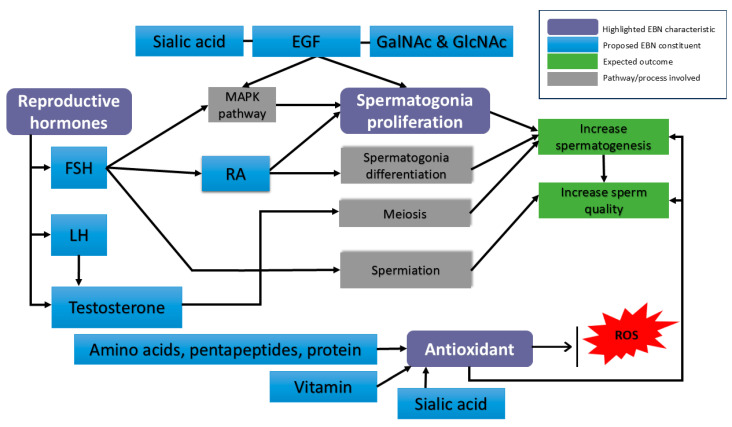
Proposed molecular mechanisms of EBN action in male reproductive physiology. Purple boxes represent the EBN characteristic. Blue boxes represent potential constituents in EBN that might benefit spermatogenesis. EGF—Epidermal growth factor; FSH—follicle-stimulating hormone; LH—luteinizing hormone; RA—retinoic acid; ROS—reactive oxygen species; MAPK pathway—mitogen-activated protein kinase.

The association between these three EBN characteristics and male reproductive function is illustrated in Figure 2. In addition, Table 2 summarizes the key molecules underlying each characteristic along with their probable effects on male reproductive outcomes.

## 4. Scientific Evidence of EBN on the Male Reproductive System

Although reports on the effects of EBN on male reproductive health are relatively sparse, the existing evidence shows promising outcomes and underscores EBN’s potential as a male fertility supplement.

### 4.1. In Vivo Studies

The presence of reproductive hormones, including testosterone, E2, progesterone, LH, FSH, and prolactin in EBN extract was first identified by Ma and Liu [12]. The biological relevance of these hormones, particularly testosterone, was demonstrated when EBN was administered at 9 mg/kg/day to castrated male rats [40]. At this dose, EBN promoted penile, prostatic, and seminal vesicle development, enhanced serum testosterone and LH secretion, and increased endothelial nitric oxide synthase expression. These observations were comparable to those in rats injected intramuscularly with testosterone propionate, leading to the conclusion that the testosterone content of EBN is the key bioactive compound responsible for the observed effects. Such findings are consistent with the established role of testosterone in regulating erectile function through nitric oxide synthase pathways in penile erectile tissue [94,95,96,97].

Beyond erectile function, another vital dimension of male fertility is semen quality. Men worldwide are increasingly experiencing low sperm counts, a condition known as oligospermia. According to the World Health Organisation (WHO), oligospermia is defined as a sperm concentration below 2 × 10^6^/mL [98]. A recent study by Jaffar et al. [64] reported that sperm concentration in adult Sprague Dawley rats increased gradually with higher doses of 250 mg/kg EBN supplementation. Furthermore, supplementation at 250 mg/kg significantly enhanced spermatogonia proliferation in rats exposed to radiofrequency electromagnetic field (RF-EMF) from a Wi-Fi router [99]. This effect was most likely mediated through hormonal regulation, particularly by FSH, rather than by modulating the c-KIT–SCF signalling pathway [99].

Complementary findings from another study showed that EBN supplementation of the same dose in Wi-Fi–exposed Sprague Dawley rat pups improved testicular oxidative stress status and sperm quality, further supporting its antioxidative role against RF-EMF–induced reproductive impairment [100]. On the same note, EBN supplementation also improved histomorphometry of the testis [101] and the hormonal profile of the male rats exposed to the RF-EMF [102].

Similarly, daily EBN supplementation at 250–1000 mg/kg for 28 days in a busulfan-induced oligospermia model significantly improved sperm concentration, seminiferous tubule diameter, germ cell height, and spermatogenesis index scores compared with busulfan-treated controls. Significantly, EBN also reduced germ cell apoptosis and upregulated p38 MAPK (MAPK14) expression, suggesting that its restorative effects are mediated through MAPK signalling [103]. Collectively, these results complement in vitro evidence and point to MAPK–PI3K–AKT pathways as central mediators of EBN’s proliferative action.

### 4.2. In Vitro Studies

A recent approach to exploring EBN’s fertility-enhancing potential has been its incorporation into commercial semen extenders. The incubation of stallion semen samples with EBN-supplemented E-Z mixin^®^ (Animal Reproduction Systems, a division of Dupree, Inc., Chino, CA, USA) extender significantly increased total motility (TM) and progressive motility (PM). It also improved sperm kinematics, including average path velocity (VAP), straight-line velocity (VSL), and curvilinear velocity (VCL). However, other parameters of sperm kinematics, namely amplitude of lateral head displacement (ALH), linearity (LIN), straightness, and beat cross frequency, were not significantly affected [104]. By contrast, supplementation of EquiPlus^®^ (Minitube, Tiefenbach, Germany) and I.N.R.A. 96^®^ (IMV Technologies Group, L’Aigle, France) extenders with EBN did not produce significant improvements in sperm motility, progressive motility, or kinematic parameters. Similar negative findings were reported when stallion semen was incubated with EBN-supplemented INRA Freeze^®^ (IMV Technologies Group, L’Aigle, France) and EquiPlus Freeze^®^ Minitube, Tiefenbach, Germany) extenders [105].

All this scientific evidence was summarised in Table 3. Taken together, these findings highlight the need for further assessment of EBN’s effects on male fertility. While the evidence to date points to promising hormonal, proliferative, and antioxidative impacts, additional studies, including well-designed clinical trials, are required to validate EBN’s therapeutic potential in improving male reproductive outcomes.

## 5. Concern and Safety of EBN on the Male Reproductive System

Even though EBN is an efficient cell proliferation promoter, the currently available scientific proof was generated in vitro. The primary concern with cell proliferation is the likelihood that EBN can encourage excessive cell growth, which would then initiate the development of malignant tissue. Nevertheless, Roh et al. [25] reported that no significant proliferation occurs in the human cancer cell lines of MCF-7 and Hep2B. Similarly, there is no significant growth of human breast adenocarcinoma cells (MCF-7), human alveolar adenocarcinoma cells (A549), human epithelial colorectal adenocarcinoma cells (Caco-2), and human colorectal carcinoma cells (HCT116) was reported [106]. In addition to in vitro testing on different cancer cell lines, in vitro SSC propagation can best validate the proliferative effect of EBN on spermatogenesis [107]. This cultivation method was successfully shown in rodent gonocytes and spermatogonia, but it is still difficult to apply to primate SSCs [108].

Besides an in vitro assessment of various cancer cell lines, EBN supplementation and its potential to cause abnormal proliferation of cells should be evaluated in an in vivo system. Intricate interactions occur between diverse niches in an in vivo system, which could lead to numerous outcomes. EBN has been linked to increased spermatogonia proliferation against Wi-Fi radiation [99] and considerable gonadotrophic cell proliferation in the pituitary gland to alleviate lead toxicity in female rats [109]. Both investigations examined in vivo cell proliferation and found no evidence of aberrant tissue growth. However, additional monitoring is necessary when alternative EBN sources, types, and doses are implemented.

Besides the monitoring of any abnormal tissue growth, the potential of EBN to mitigate the decline in male reproductive status can be achieved by using a few well-established models of male fertility in rodents. These models include the busulfan-induced model [110,111], cimetidine-induced model [112], and nicotine-induced model [113]. All of these in vivo models were proven to decrease semen parameters such as sperm motility, sperm morphology, and sperm count. It also disturbs sexual hormones, increases testicular oxidative stress, and alters the histological structure of the seminiferous tubule [111,112,113].

The hormonal content of EBN may also need to be evaluated. The most noticeable hormonal properties of EBN are its estrogenic properties [114,115]. Estrogen is thought to play a more dominant role in females than in the male reproductive system. However, the primary form of estrogen, E2, also has a significant effect on male sexual function. For instance, the libido of a male depends on the balance between testosterone and E2 (T: E). An increase in E2 level leading to a low T: E ratio may negatively affect libido [63,116] and cause erectile dysfunction [117,118].

EBN also contains prolactin, which is another well-known female hormone that is crucial for milk production during lactation [119]. Although prolactin receptors are recognised in interstitial cells, Sertoli cells, and germ cells, their role in maintaining the male reproductive system is still debatable [62]. However, its excessive level is known to contribute to male infertility by impairing sperm production [120], causing erectile dysfunction and a decrease in libido [121].

Given that the reproductive hormone strictly regulates all the reproductive functions in males, any additional external source of hormones may alter the normal male physiological reproductive function. Therefore, monitoring the hormonal content of EBN, particularly its estrogenic action, is essential so that the beneficial effects of EBN on the male reproductive system can be highlighted rather than its adverse effects.

Toxicity evaluations of EBN supplementation are sparse. To date, Hou et al. [27] monitored the effect of 0.3, 0.6, and 1.2 g/kg on a menopausal animal model. Their study reported that all doses cause no changes in the levels of liver alanine transaminase (ALT), alkaline phosphatase (ALP), and gamma-glutamyl transferase (GGT). Urea and creatinine also show no significant changes in monitoring the kidney function upon receiving the doses. No reproductive toxicity testing of EBN has ever been performed. Given that EBN contains numerous bioactive compounds, high levels of nitrate and nitrite [122], heavy metals [123], and adulteration [124], this issue needs to be addressed immediately.

To address this concern, Malaysian regulations have established specific maximum limits for lead, arsenic, and mercury in raw cleaned edible bird’s nest (EBN). In contrast, other jurisdictions generally apply broader food-group limits. Nevertheless, studies have reported substantial variability in contaminant levels among EBN samples, with some exceeding these national thresholds [125]. These findings highlight the necessity for continuous surveillance, standardised processing practices, and stringent quality assurance measures to ensure the safety and regulatory compliance of EBN intended for human consumption. This concern has been listed in Table 4.

## 6. Future Perspective of EBN on the Male Reproductive System

According to the available evidence and safety concerns regarding EBN, particularly as it relates to the male reproductive system, there is currently a dearth of information regarding the effects of EBN on male infertility. To date, EBN’s effects are limited to its ability to treat erectile dysfunction, improve several sperm parameters, and preserve spermatogonia proliferation against Wi-Fi exposure. Nevertheless, the data of each study remains limited.

Extensive research still needs to be conducted, particularly to determine the potential of EBN in addressing issues with male infertility. Although a study by Jaffar et al. [64] indicated an improvement in sperm quality, the impact of EBN should be further assessed by using known infertile models. An induced animal model can be used to cause infertility problems such as oligozoospermia, asthenozoospermia, or teratozoospermia. Work involving such models will contribute to the knowledge of EBN’s potential to address male infertility issues.

**Figure 3:**
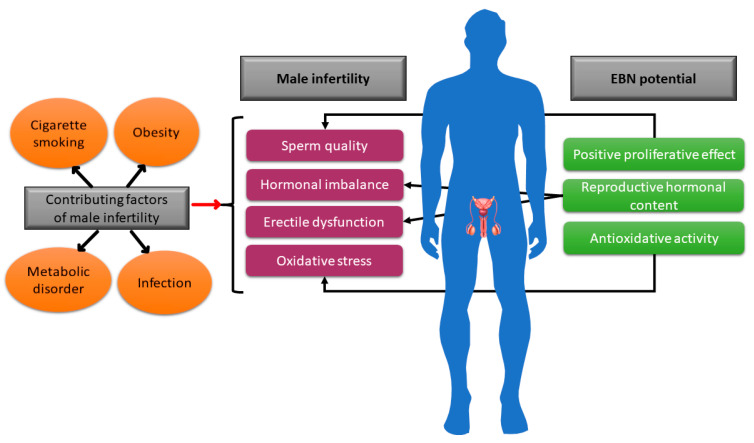
Depiction of a few male infertility contributing factors and the possible action of EBN to augment the infertility status in males.

Future research should assess the effectiveness of EBN in reducing oxidative stress in male infertility cases. The male reproductive system can experience oxidative stress in a variety of ways. One of them is to adopt elements of contemporary lifestyles like cigarette smoking, consuming a high-fat diet to induce obesity, or developing metabolic disorders such as diabetes (Figure 3). These elements contribute to male infertility.

No study has yet evaluated how the traditional EBN claim affects male libido. Male libido may be increased by several key reproductive hormones found in EBN, thereby supporting the conventional superstition. This EBN characteristic might be helpful to attenuate male infertility linked to hormonal imbalance, which frequently occurs in obese males [128]. However, monitoring of all reproductive hormonal levels in EBN is necessary because of concerns regarding its effects on the male reproductive system. Furthermore, an evaluation of EBN toxicity, particularly about the reproductive system, is urgently needed.

Finally, a comparison of EBN and the present male infertility supplement regimen offered by fertility clinics is an interesting topic for future research. The current method of treating male infertility involves using antioxidant supplements, such as Profortil [129], coenzyme Q10 [84], and zinc [130]. Given that EBN has three or possibly more bioactive compounds in a single supplement that is essential for the reproductive system, it may become an effective food supplement for male fertility.

## Figures and Tables

**Table 1:** Composition of EBN and its potential benefit to the male reproductive system.

Component	Content	Reference	Potential Benefit
Proximate analysis (%):		[41]	Supplies macronutrients essential for energy metabolism and maintenance of testicular function.
Moisture	12.28–16.62		
Ash	1.85–10.70		
Protein	54.29–60.59		
Fat	0.43–1.19		
Carbohydrate	18.98–26.32		
Amino acids (g/100 g):		[41]	Support spermatogonia proliferation and testicular matrix maintenance Serve as a source for antioxidant protection
Essential amino acids			
Lysine	1.74–1.93		
Threonine	3.09–3.48		
Leucine	2.88–3.29		
Phenylalanine	2.62–3.01		
Valine	3.98–4.62		
Isoleucine	1.40–1.58		
Histidine	1.92–2.38		
Methionine	0.03–0.05		
Non-essential amino acids			
Serine	4.97–5.68		
Aspartic acid	4.46–5.07		
Glutamic acid	3.92–4.27		
Proline	3.43–3.91		
Alanine	1.76–2.95		
Arginine	3.12–3.41		
Glycine	2.10–2.32		
Hydroxyproline	0.32–0.39		
Tyrosine	3.07–3.54		
Fatty acid (%):		[9]	Maintain sperm membrane fluidity Act as a source for antioxidant protection
Palmitic C16:0	23–26		
Steric C18:0	26–29		
Linoleic C18:1	22		
Linolenic C18:2	26		
Triacylglycerol (%):		[9]	Provides lipid energy reserves Supports sperm membrane stability Acts as an antioxidant defence
Palmitic-Palmitic-Steric	14–16		
Steric-Steric-Linoleic	13–15		
Palmitic-Linolenic-Linolenic	18–19		
Monoglycerides	27–31		
Diglycerides	21–26		
Vitamin:		[42]	Vitamin A regulates spermatogonia differentiation Vitamin D supports androgen production Vitamin C contributes to oxidative defence
A (IU/mg)	2.57–30.40		
D (IU/mg)	60.00–1280.00		
C (mg/100 g)	0.12–29.30		
Elemental analysis (mg/kg dry matter):		[43]	Supports electrolyte balance necessary for membrane potential regulation and sperm motility Calcium ion regulates sperm motility and acrosome reaction Magnesium and zinc are known cofactors in spermatogenesis and testosterone biosynthesis Zinc serves as an antioxidant source
Sodium	16,095–17,937		
Potassium	647–898		
Calcium	6605–19,577		
Magnesium	1790–2818		
Phosphorus	0.27–0.31		
Iron	22.02–28.76		
Zinc	4.18–8.02		
Copper	5.99–6.31		
Lead	0.194–0.233		
Cadmium	0.004–0.007		
Mercury	0.084–0.099		
Arsenic	0.075–0.088		
Hormone:		[12]	Detected in trace amounts Physiologically relevant hormones in maintaining overall male reproductive function
Testosterone (ng/g)	4.293–12.148		
Estradiol (pg/g)	802.333–906.086		
Progesterone (ng/g)	24.966–37.724		
LH (mIU/g)	1.420–11.167		
FSH (mIU/g)	0–0.149		
Prolactin (ng/g)	0–0.392		

**Table 2:** Summary of EBN characteristics, key molecules, and benefits for male reproductive function.

Characteristics of EBN	Key Molecules Associated with the Characteristic of EBN	Probable Effect on Male Reproductive Function
Proliferative effect	EGF Sialic acid GalNAc GlcNAc	Stimulates mitosis of spermatogonia stem cells (SSC) via MAPK signalling in the seminiferous tubules, thereby enhancing spermatogenesis outcomes.
Reproductive hormonal content	FSH LH Testosterone E2 Prolactin Progesterone	Enhance libido and regulate hormone-dependent spermatogenesis in the testis. Therefore, contributes to overall male reproductive performance.
Antioxidant activity	Amino acids: Histidine Proline Phenylalanine Tryptophan Pentapeptides: Pro-Phe-His-Pro-Tyr Leu-Leu-Gly-Asp-Pro Protein: Ovotransferrin Lactoferrin Carbohydrate moiety: Sialic acid Vitamin: A C D	Scavenges reactive oxygen species (ROS), protects spermatozoa from oxidative damage, enhances antioxidant defence in testicular tissue, and improves sperm quality and function under oxidative stress conditions.

**Table 3:** Scientific evidence of EBN effects on the male reproductive system.

EBN Dosage and Duration	Route of Administration	Study Model	Findings	References
In vivo				
1 mg/kg/day, 3 mg/kg/day, 9 mg/kg/day for 10 days	Intragastric	Castrated rats	Significantly increased prostate and seminal vesicle index, eNOS expression, testosterone and LH	[40]
10 mg/kg/day, 50 mg/kg/day, 250 mg/kg/day for 60 days	Oral (EBN enriched mouse pellet)	Healthy rats	EBN supplementation at a dose of 250 mg/kg caused a significant increase in sperm concentration, percentage of sperm motility, as well as FSH and LH levels compared to the 10 mg/kg dose. There was a dose-dependent increase in testosterone level, but it was not significant between groups.	[64]
250 mg/kg/day for 60 days	Oral (EBN enriched mouse pellet)	Wi-Fi induced testicular damage in rat pups	Significant increase in spermatogonia mitotic status Significant increase in serum FSH level	[99]
250 mg/kg/day for 60 days	Oral (EBN enriched mouse pellet)	Wi-Fi induced testicular damage rat pups	Significantly reduced testicular total oxidant status and 8-hydroxy-2′-deoxyguanosine expression Significant increase in sperm chromatin integrity, morphology, and concentration	[100]
250 mg/kg/day for 60 days	Oral (EBN enriched mouse pellet)	Wi-Fi-induced testicular damage in adult rats	EBN supplementation restored serum FSH and testosterone levels Increased serum LH levels and the testosterone/E2 ratio Normalised mRNA ERα expression EBN supplementation increased sperm concentration in Wi-Fi-exposed rats	[102]
250 mg/kg/day, 500 mg/kg/day, 1000 mg/kg/day for 28 days	Oral (EBN enriched mouse pellet)	Busulfan-induced oligospermia	EBN supplementation significantly increased sperm concentration, spermatogenesis index scoring, and germ cell height Significantly increased gene expression of p38 and MAPK14 EBN supplementation also reduced apoptosis in the oligospermia group in a dose-dependent manner	[103]
250 mg/kg/day for 60 days	Oral (EBN enriched mouse pellet)	Wi-Fi-induced testicular damage rat pups	EBN supplementation preserved seminiferous tubule cells, maintaining their standard and intact structure. It also significantly increased seminiferous tubule diameter compared with the Wi-Fi-exposed group that did not receive EBN supplementation.	[101]
In vitro				
0%, 0.12%, 0.24%, 0.24% + seminal plasma for 0 h, 24 h and 48 h	EBN-supplemented E-Z mixin^®^ extender	Chilled Arabian stallion semen	0.12% and 0.24% EBN-supplemented extender significantly increased sperm total motility and progressive motility. It also improved sperm kinematics	[104]
0%, 0.12%, 0.24%, for 0 h, 24 h and 48 h	EBN-supplemented EquiPlus^®^ and INRA 96^®^ extenders	Chilled and post-thawed cryopreserved Arabian stallion spermatozoa	No significant improvements in sperm motility, progressive motility, No improvement in the sperm kinematic parameters	[105]

**Table 4:** Toxicological issues, current regulatory limits, and research needs for EBN.

Contaminant/ Constituent	Regulatory Limit by Malaysian Food Act 1983 [126] (Raw Cleaned EBN)	Regulatory Limit Set before Exporting Raw, Uncleaned EBN [127]	Toxicological Concerns for Human Health	Key Research Needs
Lead	0.30 mg/kg	≤2 mg/kg	Neurotoxicity, especially in children Reproductive toxicity, reduced sperm quality, endocrine disruption	EBN reproductive toxicity studies
Arsenic	0.15 mg/kg	≤1 mg/kg	Inorganic arsenic is carcinogenic Chronic exposure affects reproductive health and sperm DNA integrity	Speciation analysis for inorganic vs. organic arsenic dietary exposure and reproductive studies.
Mercury	0.07 mg/kg	≤1 mg/kg	Neurotoxicity Reproductive toxicity Decreased semen quality	Measure total/methylmercury in EBN Determine sources and model human intake
Cadmium	N/A	≤1 mg/kg	Kidney toxicity, testicular damage, and reduced sperm quality.	Large-scale surveillance for cadmium Reproductive risk modelling
Trace elements	N/A	N/A	Essential at low levels (e.g., Zn supports spermatogenesis), whereas excess can be toxic.	Report complete elemental profiles Assess cumulative exposure and reproductive relevance.
Microbial/ Mycotoxins	Nitrite ≤ 30 mg/kg [125]	*Escherichia coli*: ≤1.0 × 10^2^ cfu/g *Salmonella enteritidis*: not detected *Salmonella typhimurium*: not detected *Salmonella gallinarum*: not detected Coagulative Positive *Staphylococcus aureus*: ≤2.5 × 10^3^ cfu/g *Listeria monocytogenes*: Not detected	Foodborne illness Nitrite and mycotoxins linked to reproductive toxicity.	Routine testing for microbial contamination and mycotoxins.
Hormonal contents	N/A	N/A	Potential endocrine effects if bioavailable at relevant doses Risk for endocrine-sensitive conditions.	Quantify hormones by LC–MS/MS Evaluation of oral bioavailability and endocrine activity

## Data Availability

No data sets were generated or analyzed during the present study.

## Abbreviations

The following abbreviations are used in this manuscript:

ABTS	2,2′-azino-bis [3-ethylbenzothiazoline-6-sulphonic acid]
AP-1	Activator protein-1 pathway
DPPH	1,1-diphenyl-2-picrylhydrazyl
EBN	Edible Bird Nest
E2	Estradiol
EGF	Epidermal growth factor
ERα	Estradiol receptors α
ERβ	Estradiol receptors β
FGF	Fibroblast growth factor
FSH	Follicle-stimulating hormone
GalNAc	N-acetylgalactosamine
GlcNAc	N-acetylglucosamine
GDNF	Glial cell line-derived neurotrophic factor
Gpx	Glutathione peroxidase
Gsr	Glutathione reductase
H_2_O_2_	Hydrogen peroxide
hADSCs	Human adipose-derived stem cells
IL-6	Interleukin-6
LC-MS	Liquid chromatography mass spectrometry
LH	Luteinizing hormone
MAPK	Mitogen-activated protein kinase
NF-κB	Nuclear factor kappa B
NANA	N-acetylneuraminic acid
ORAC	Oxygen radical absorbance capacity
PI3K	Phosphoinositide 3-kinase
RA	Retinoic acid
RF-EMF	Radiofrequency electromagnetic field
ROS	Reactive oxygen species
SOD	Superoxide dismutase
SSCs	Spermatogonia stem cells
VEGF	Vascular endothelial growth factor
